# Eupatilin exerts neuroprotective effects in mice with transient focal cerebral ischemia by reducing microglial activation

**DOI:** 10.1371/journal.pone.0171479

**Published:** 2017-02-08

**Authors:** Arjun Sapkota, Bhakta Prasad Gaire, Kyu Suk Cho, Se Jin Jeon, Oh Wook Kwon, Dae Sik Jang, Sun Yeou Kim, Jong Hoon Ryu, Ji Woong Choi

**Affiliations:** 1 College of Pharmacy and Gachon Institute of Pharmaceutical Sciences, Gachon University, Incheon, Republic of Korea; 2 Department of Oriental Pharmaceutical Science, College of Pharmacy, Kyung Hee University, Seoul, Republic of Korea; Universita degli Studi di Napoli Federico II, ITALY

## Abstract

Microglial activation and its-driven neuroinflammation are characteristic pathogenetic features of neurodiseases, including focal cerebral ischemia. The *Artemisia asiatica* (Asteraceae) extract and its active component, eupatilin, are well-known to reduce inflammatory responses. But the therapeutic potential of eupatilin against focal cerebral ischemia is not known, along with its anti-inflammatory activities on activated microglia. In this study, we investigated the neuroprotective effect of eupatilin on focal cerebral ischemia through its anti-inflammation, particularly on activated microglia, employing a transient middle cerebral artery occlusion/reperfusion (tMCAO), combined with lipopolysaccharide-stimulated BV2 microglia. Eupatilin exerted anti-inflammatory responses in activated BV2 microglia, in which it reduced secretion of well-known inflammatory markers, including nitrite, IL-6, TNF-α, and PGE_2_, in a concentration-dependent manner. These observed *in vitro* effects of eupatilin led to *in vivo* neuroprotection against focal cerebral ischemia. Oral administration of eupatilin (10 mg/kg) in a therapeutic paradigm significantly reduced brain infarction and improved neurological functions in tMCAO-challenged mice. The same benefit was also observed when eupatilin was given even within 5 hours after MCAO induction. In addition, the neuroprotective effects of a single administration of eupatilin (10 mg/kg) immediately after tMCAO challenge persisted up to 3 days after tMCAO. Eupatilin administration reduced the number of Iba1-immunopositive cells across ischemic brain and induced their morphological changes from amoeboid into ramified in the ischemic core, which was accompanied with reduced microglial proliferation in ischemic brain. Eupatilin suppressed NF-κB signaling activities in ischemic brain by reducing IKKα/β phosphorylation, IκBα phosphorylation, and IκBα degradation. Overall, these data indicate that eupatilin is a neuroprotective agent against focal cerebral ischemia through the reduction of microglial activation.

## Introduction

*Artemisia asiatica* Nakai (Asteraceae; *A*. Nakai) is a well-known traditional medicine in Asia to treat the inflammation-relevant diseases [1,2]. The therapeutic potential of *A*. Nakai for various diseases, including gastric ulcer, liver injury, and pancreatic damage, is related to its anti-inflammatory effects [1–5], likely by regulating nuclear factor κB (NF-κB) pathways [6]. These translational researches led to the clear success, Stillen^™^ that is the formulated extract of *A*. Nakai and currently used to treat gastric mucosal ulcers in South Korea. Eupatilin (5,7-dihydroxy-3’,4’,6’-trimethoxy flavone), a main flavone component from *A*. Nakai extract, has been believed to exert pharmacological activities of the extract, including anti-oxidative [7,8] and anti-inflammatory [9–12] activities.

The above beneficial effects of *A*. Nakai extracts and eupatilin have been demonstrated mostly outside of the brain. However, there is a high possibility that they work as neuroprotective agents against brain diseases due to their pharmacological potential on inflammation, a core pathogenesis in diverse brain diseases including cerebral ischemia. Despite these medically relevant properties, there have been only a few reports implicating possible neuroprotection by *A*. Nakai extracts and eupatilin. In mice subjected to the forced swimming test, Stillen^™^ induced anti-depression-relevant effects where it increased levels of 5-hydroxytryptamine, brain-derived neurotrophic factor, and estrogen receptor-β and decreased levels of proinflammatory cytokines in the hippocampus [13]. In transient global ischemia model, eupatilin attenuated cell death of hippocampal neurons, along with an enhancement of Akt phosphorylation [14]. Additionally, a prior *in vitro* study has demonstrated that *A*. Nakai extract reduces inflammatory responses in activated microglia [15], a key pathogenesis in diverse brain diseases [16,17]. However, there is no report on the similar effect of eupatilin on microglia and the therapeutic potential for microglial activation-relevant brain diseases, such as focal cerebral ischemia that is caused by an insufficient blood supply to the particular region of the brain, leading to the damage of that area and surrounding regions [18]. More importantly, the attenuation of microglial activation has been considered as an important therapeutic strategy against focal cerebral ischemia [16–18]. Therefore, it is possible that eupatilin reduces microglial activation and thereby attenuates brain damage in focal cerebral ischemia.

Here, we have assessed whether eupatilin reduces microglial activation *in vitro* using lipopolysaccharide (LPS)-stimulated murine BV2 microglia. We also have assessed the neuroprotective activities of eupatilin *in vivo* against focal cerebral ischemia utilizing mice subjected to transient middle cerebral artery occlusion/reperfusion (“tMCAO”), a representative animal model of focal cerebral ischemia, particularly in view of its effects on microglial activation in ischemic brain.

## Materials and methods

### Materials

Eupatilin with HPLC purity of about 97% was prepared from *Artemisia* species. In brief, the leaves and young stem of *Artemisia argyi* Lev. et Vant. were obtained from Richwood Co. (Seoul, Korea) in June, 2014. A voucher specimen (no. 2014-ARAR01) has been deposited in the Lab. of Natural Product Medicine, College of Pharmacy, Kyung Hee University, Republic of Korea. The dried plant materials (1 kg) were extracted 3 times with MeOH (9.4 L) at room temperature, and then the solution was evaporated under vacuo. The MeOH extract (142 g) was subjected to Diaion HP-20 column chromatography (6.3 × 49.5 cm) and eluted with a stepwise gradient of MeOH-H_2_O system (3:2 to MeOH 100%) to yield 6 subfractions (Fr-1 ~ Fr-6). Eupatilin (718.9 mg) was isolated by recrystallization in MeOH from Fr-5 (7.91 g). 3-Methyl-1-phenyl-2-pyrazolin-5-one (Edaravone), 2, 3, 5-triphenyltetrazolium (TTC), 3, 3′-diaminobenzidine tetrahydrochloride (DAB), lipopolysaccharide (LPS), bromodeoxyuridine (BrdU), and L-NG-Monomethyl-L-arginine (L-NMMA) were purchased from Sigma-Aldrich (St. Louis, MO, USA). Silicon (Variotime^®^) or Zoletil 50^®^ were obtained from Heraeus Kulzer GmbH (Wehrheim, Germany) or Virbac Laboratories (Carros, France). Antibodies against rabbit ionized calcium-binding adapter molecule 1 (Iba1) and rat BrdU were purchased from Abcam (Cambridge, UK). Antibodies against rabbit glial fibrillary acidic protein (GFAP) and rabbit NF-κB p65 were purchased from Invitrogen (Carlsbad, USA) and Santa Cruz Biotechnology (Santa Cruz, USA), respectively. Antibodies against rabbit phospho-IκB kinase α/β (p-IKKα/β (Ser176/Ser180)), rabbit phospho-IκB α (p-IκBα (Ser32)), rabbit IκB α (IκBα), and mouse β-actin were purchased from Cell signaling (Hitchin, UK). Avidin-biotin-peroxidase complex (ABC) kit and VECTASHIELD^®^ were purchased from Vector Laboratories (Burlingame, CA, USA). Rabbit polyclonal anti-4-hydroxynonenal (4-HNE) and Cy3-conjugated secondary antibodies were purchased from Bioss (Freiburg, Germany) and Jackson ImmunoResearch (West Grove, USA). The AF488-conjugated secondary antibody was from Invitrogen (Carlsbad, USA). Competitive enzyme immunoassay kits for interleukin-6 (IL-6), tumor necrosis factor-α (TNF-α), and prostaglandin E2 (PGE_2_) were obtained from R&D systems (Minneapolis, USA).

### *In vitro* anti-inflammatory assays

The murine microglial BV2 cells were maintained in Dulbecco’s modified Eagle’s medium (DMEM) supplemented with 10% fetal bovine serum, and 100 U/ml penicillin, and 100 μg/ml streptomycin.

In order to measure NO production, BV-2 cells were plated into 96-well plate (3 × 10^4^ cells/well) and treated with 100 ng/ml of LPS in the presence or absence of eupatilin for 24 h. Nitrite (NO_2_), a soluble oxidation product of NO, in the culture media was detected using the Griess reaction, as previously reported [17]. To measure PGE_2_, TNF-α, and IL-6, at the end of the treatment as above, the conditioned medium were obtained from BV-2 cells grown on 24-well plate (3 × 10^5^ cells/well). Concentrations of PGE_2_, TNF-α, and IL-6 in the conditioned medium were measured by competitive enzyme immunoassay kits according to the manufacturer’s protocols.

### Challenge of tMCAO and drug administration

All animal experimental procedures were performed in accordance with approved animal protocols by the Institutional Animal Care and Use Committee at Lee Gil Ya Cancer and Diabetes Institute (LCDI), Gachon University, Korea (# of approved animal protocol: LCDI-2013-0074). Male ICR mice (7 weeks old, 28–32 g; Orient Bio, SeongNam, Korea) were subjected to tMCAO (90 min of occlusion) as previously described [17]. Briefly, mice were anesthetized with isoflurane (3% for induction and 1.5% for maintenance) in N_2_O:O_2_ (3:1) and MCAO was induced by inserting a 9-mm-long 5–0 nylon monofilament coated with silicon from the right common carotid artery (CCA) bifurcation to the MCA. The monofilament was withdrawn 90 min after occlusion under anesthesia to restore blood flow. Sham group was subjected to the same surgical procedure, except for the occlusion. Mice that were challenged with tMCAO were randomly divided into the vehicle-treated, the eupatilin-treated, or the edaravone-treated groups. Eupatilin or edaravone was dissolved in 10% Tween 80 (vehicle). Each group was administered vehicle (10% Tween 80, *p*.*o*.), eupatilin (1, 3, 10 mg/kg, *p*.*o*.), or edaravone (3 mg/kg, *p*.*o*.) immediately after reperfusion. Alternatively, eupatilin (10 mg/kg) was orally administered to mice 5 hours after MCAO induction. Edaravone (Radicut^®^, Mitsubishi Tanabe Pharma Co.), a well-known therapeutic agent for the acute cerebral ischemia in Japan [19,20], was used as a positive control. Ten to fifteen mice were used per each experimental group to determine brain infarction and neurological dysfunction, and 4 to 6 mice were used per each group to perform histological or biochemical analysis. After tMCAO challenge, mice were housed 3 per cage with moist food and soft bedding materials to reduce suffering. Mice were monitored by visual inspection and body weight measurement every 12 hours following tMCAO till the experimental endpoints. In this study, there was no mouse that was processed for the early euthanasia (by CO_2_ exposure) determined by, in general, abnormal illness (i.e., no movement) or reduced body weight (10% loss in a day). In total, four mice died within 12 hours after tMCAO challenge possibly due to the severe ischemic damage as follows. One mouse of each experimental group (tMCAO+vehicle, tMCAO+1 mg/kg eupatilin, and tMCAO+3 mg/kg eupatilin) died before the measurement of brain infarction 1 day after tMCAO challenge. In addition, one mouse of the vehicle-treated group to determine brain damage 3 days after tMCAO challenge was dead.

### Measurement of infarction volume and functional neurological deficit score

Several parameters regarding modified neurological severity score (mNSS) was determined at the end of experiments using well-known criteria. The total mNSS was obtained by the summation of partial scores of general (posture, pinna reflex, corneal reflex, epileptic behaviors etc.), focal (body symmetry, gait disorder, circling behaviors, flexion of limbs etc.), and balance and sensory deficit with a maximum of 18 points and minimum of 0 in normal mice as previously described [21]. After obtaining the neurological score, mice were euthanized by CO_2_ exposure, and their brains were quickly removed and sectioned into 2 mm thick coronal sections. Brain infarct volume was determined using TTC-stained brain slices as previously described [17].

### Histological assessment

#### Tissue preparation

Brain samples for histological assessment were obtained 1 or 3 days after reperfusion. Mice were anesthetized with a mixture of Zoletil 50^®^ (10 mg/kg, *i*.*m*.) and Rompum^®^ (3 mg/kg, *i*.*m*.) and perfused transcardially with ice-cold phosphate buffered saline (PBS) followed by 4% paraformaldehyde. Removed brains were post-fixed overnight with the same fixative, immersed in 30% sucrose solution, embedded in Tissue-Tek Optimal Cutting Temperature (OCT) compound, and frozen on dry ice. Brain sections (20 μm) were prepared using a J4800AMNZ cryostat microtome (Thermo Fisher Scientific, Dreieich, Germany) and used for histological analysis.

#### Fluoro Jade-B staining

Extent of neural cell death after tMCAO challenge was determined by Fluoro-Jade B (FJB) staining. Cryostat sections were washed with water, immersed in an alcohol series (100% ethanol for 3 min, 70% ethanol for 1 min, and 30% ethanol for 1 min), and rinsed with water for 1 min. Sections were stained with 0.001% FJB in 0.1% acetic acid solution for 30 min after oxidation by soaking them in 0.06% potassium permanganate for 15 min. Stained sections were rinsed with water, dried on the slide warmer, dehydrated with xylene, and cover-slipped with Entellan^®^ mounting medium (Merck, Germany). Fluorescent images were collected using a fluorescent microscope (BX53, Olympus, Tokyo, Japan) equipped with a DP72 camera (Olympus).

#### Immunohistochemistry

Immunohistochemical analyses were performed on cryostat sections to detect microglial activation and lipid peroxidation using antibodies against Iba1 and 4-HNE, respectively. Brain sections were treated with 1% H_2_O_2_, blocked with 1% fetal bovine serum (FBS) in 0.3% TritonX-100, and labeled with rabbit anti-Iba1 (1:500) or rabbit anti-4-HNE (1:500) antibody overnight at 4°C. The sections were further reacted with secondary biotinylated anti-rabbit IgG antibody (1:200), followed by labeling with avidin/biotin complex. Labeled sections were developed with an exposure to 0.02% DAB and 0.01% H_2_O_2_ for 2 min, rinsed three times with PBS, and dehydrated through an ethanol and xylene series. Colored images were obtained using the BX53 microscope. For the quantitative analysis, the number of Iba1^+^ cells was counted in three different areas of both the periischemic and the ischemic core regions of each section and expressed as the total numbers of Iba1^+^ cells per mm^2^ area of each region.

In order to determine where NF-κB pathway is activated after tMCAO challenge, cryostat sections were processed for double immunolabeling using antibodies against NF-κB p65, Iba1 (for activated microglia), and GFAP (for activated astrocytes). Sections were incubated with TRIS-EDTA solution at 100°C for 30 min for antigen retrieval, blocked with 1% FBS in 0.3% Triton X-100, and labeled with rabbit NF-κB p65 (1:50) antibody overnight at 4°C. Sections were labeled with a biotinylated secondary antibody (1:200) followed by incubation with avidin/biotin complex (ABC, 1:100). Signals were visualized with DAB staining (0.02% DAB and 0.01% H_2_O_2_ for 2 min). Stained sections were washed with PBS (3×5 min), blocked, and incubated with primary antibodies against Iba1 (1:500) or GFAP (1:500) overnight at 4°C. Sections were labeled with appropriate secondary antibodies conjugated with Cy3 (1:1000). Colored and fluorescent images were obtained using the BX53 microscope.

#### Bromodeoxyuridine (BrdU) immunofluorescence

Double immunofluorescence for BrdU and Iba1 was used to determine microglial proliferation in the ischemic brain. Mice were treated with BrdU (50 mg/kg, *i*.*p*., for 2 days after tMCAO challenge) twice daily at a 12 h interval. The first BrdU injection was performed 12 hours after tMCAO and the final injection was done 12 h prior to brain sampling (3 days after tMCAO).

Cryostat brain sections (20 μm) were incubated with HCl (2 N) at 37°C, neutralized with borate buffer (0.1 M, pH 8.5) for 3 × 15 min, and blocked with 1% FBS in 0.3% Triton X-100. Sections were labeled with primary antibodies against BrdU (1:200) and Iba1 (1:500) overnight at 4°C and further labeled with secondary antibodies conjugated with Cy3 (1:1000) and AF488 (1:1000), respectively. Fluorescent images were taken with laser scanning confocal microscopy (Eclipse A1 Plus, Nikon, Japan). The number of Iba1 and BrdU double-immunopositive cells were counted in three different region of the penumbra and expressed as the total number of double-immunopositive cells per mm^2^ of the penumbral region.

### Western blot analysis

Brain sample for Western blot assessment were obtained 1 day after reperfusion. Mice were transcardially perfused with ice-cold PBS and ipsilateral brain hemispheres were homogenized using N-PER neuronal protein extraction reagent (Thermo Scientific, IL, USA). Brain lysates containing 20 μg of total protein was separated by 10% SDS-PAGE and electrically transferred to nitrocellulose membranes. The membranes were blocked with 0.1% polyvinyl alcohol in Tris-buffered saline (TBS, pH 7.6) containing 0.2% Tween 20 for 30 min. The membranes were incubated with primary antibodies against rabbit p-IKKα/β (1:1000), rabbit p-IκBα (1:1000), rabbit IκBα (1:1000), rabbit 4-HNE (1:1000), mouse α-tubulin (1:5000), and mouse β-actin (1:5000), respectively, overnight at 4°C, followed by the further incubation with appropriate peroxidase-conjugated secondary antibodies (Invitrogen) for 2 h at room temperature. Specific bands were detected using the ECL western blotting substrate (Thermo Scientific) and exposed to LAS-4000 image detection system (Fuji, Japan).

### Statistical analysis

Values are presented as the mean ± S.E.M. Statistical significance was set at *p* < 0.05 by analyses using one way ANOVA followed by Tukey’s range test (*in vitro* system) and Newman-Keuls post-hoc test (*in vivo* system) for multiple comparisons. The comparisons between two groups were performed using the Mann-Whitney test.

## Results

### Eupatilin reduces proinflammatory responses in LPS-stimulated BV2 microglia

**Fig 1:**
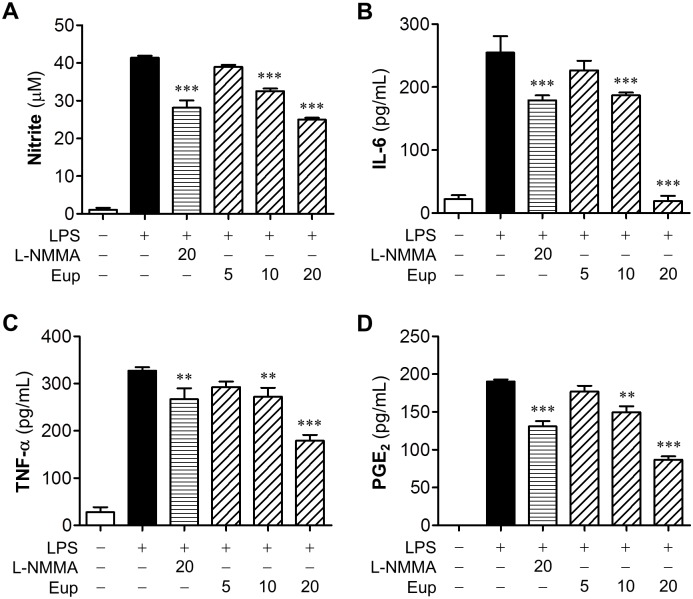
Eupatilin reduces inflammatory responses in LPS-stimulated microglia. Effects of eupatilin on levels of nitrite (**A**), IL-6 (**B**), TNF-α (**C**), and PGE_2_ (**D**) were determined in the conditioned medium from LPS (100 ng/ml, 24 h)-stimulated BV2 microglia in the presence or absence of eupatilin at different concentrations (5, 10, and 20 μM). L-NMMA (20 μM) was used as a positive control. n = 3 per group. **P<0.01 and ***P<0.001, versus the vehicle-treated LPS group (LPS+veh).

To investigate effects of eupatilin on microglial activation, we determined production of proinflammatory mediators in LPS-stimulated BV2 cells. Eupatilin reduced NO production in activated microglia to 78.5% or 60.3% at 10 or 20 μM, compared with vehicle-treated cells (Fig 1A). Eupatilin at 20 μM was more effective than a positive control, L-NMMA (20 μM) (Fig 1A). Eupatilin also concentration-dependently reduced the production of other proinflammatory mediators such as PGE_2_, TNF-α, and IL-6 (Fig 1B–1D), all of which are well-known markers for microglial activation. These results demonstrated that eupatilin reduced inflammatory responses in activated microglia.

### Eupatilin reduces brain damage in tMCAO-challenged mice

**Fig 2:**
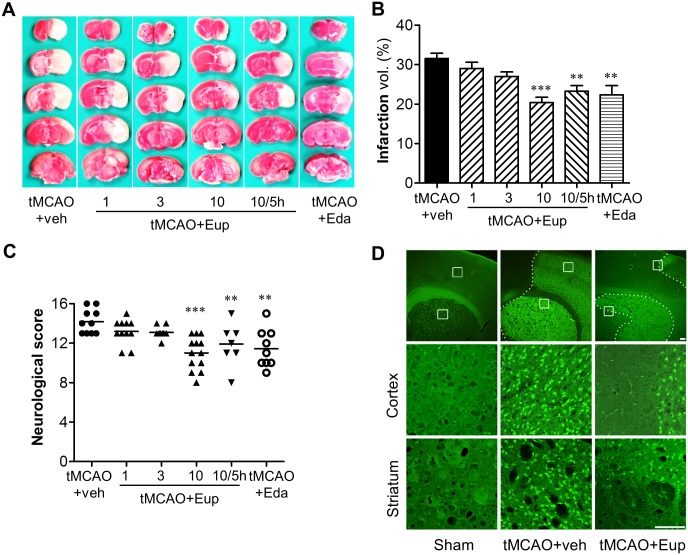
Eupatilin reduces brain damage in tMCAO-challenged mice. Mice were challenged with tMCAO and eupatilin (Eup: 1, 3, and 10 mg/kg, *p*.*o*.) was given to mice immediately after tMCAO. Alternatively, 10 mg/kg eupatilin was given to mice 5 hours after MCAO induction. Effects of eupatilin on brain infarct volume (**A and B**), neurological function (**C**), and neural cell death (**D**) were assessed 22 h after reperfusion. Edaravone (Eda, 3 mg/kg, *p*.*o*.) was used as a positive control. (**A**) Representative images of TTC-stained brain slices indicating brain infarction. (**B**) Quantification of infarct volume. (**C**) Neurological score reflecting neurological functions. n = 10~15 per group. **P<0.01 and ***P<0.001 versus the vehicle-treated tMCAO group (tMCAO+veh). (**D**) Effects of eupatilin (Eup, 10 mg/kg, *p*.*o*.) administration immediately after tMCAO on neural cell death. Representative images of FJB-stained sections. Diagram boxes display the cerebral area where the images in middle and bottom panels were acquired. Dashed lines indicate the lesion site. Scale bars, 200 μm (top panels) and 50 μm (middle and bottom panels).

**Fig 3:**
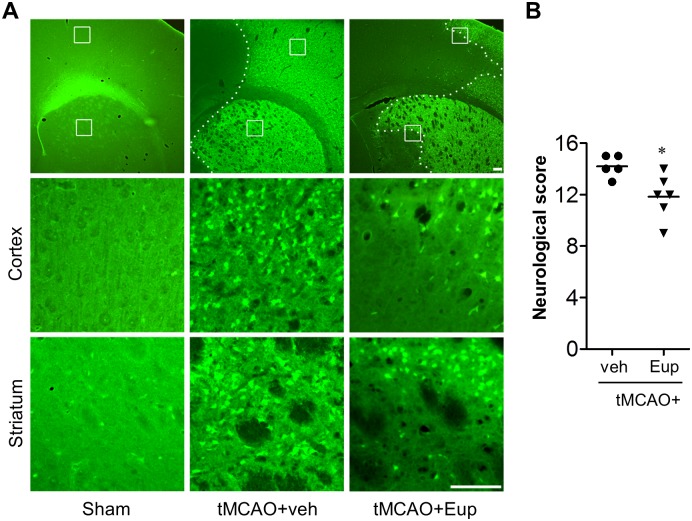
Eupatilin reduces neural cell death and improves neurological function 3 days after tMCAO-challenge. Mice were challenged with tMCAO and effects of eupatilin (Eup, 10 mg/kg, a single administration, *p*.*o*.) on neural cell death and neurological function were assessed 3 days after tMCAO. (**A**) Representative images of FJB-stained sections (n = 5 per group). Diagram boxes display the cerebral area where the images in middle and bottom panels were acquired. Dashed lines indicate the lesion site. Scale bars, 200 μm (top panels) and 50 μm (middle and bottom panels). (**B**) Quantification of neurological score reflecting neurological functions. n = 5~6 per group. *P<0.05 versus the vehicle-treated tMCAO group (tMCAO+veh).

Our previous study employing a model of a transient global ischemia demonstrated that eupatilin (10 mg/kg, *p*.*o*.) significantly reduced hippocampal neuronal death [14] and the current *in vitro* data showed a reduced microglial activation with eupatilin, both of which raises the possibility of its neuroprotective action in focal cerebral ischemia. To verify this, we determined the neuroprotective effects of eupatilin *in vivo* using tMCAO-challenged mice. Eupatilin given orally (10 mg/kg) immediately after reperfusion significantly decreased brain infarct volume to 35.3% compared with the vehicle-treated group (Fig 2A and 2B; the value of infarcted area in the vehicle- or eupatilin (10 mg/kg)-administered tMCAO group was 31.57% or 20.42%). The protective extent of eupatilin was superior to that of the positive control, edaravone that produced 22.34% of infarction (Fig 2A and 2B). Eupatilin also improved neurological function by 22.42% compared with the vehicle-administered tMCAO group (the mNSS of the vehicle- or eupatilin (10 mg/kg)-administered group was 14.18 ± 0.35 or 11.00 ± 0.41) (Fig 2C). However, lower doses (1 and 3 mg/kg) did not achieve significant protection against ischemic damage even with a slight reduction in brain infarction and neurological dysfunction (Fig 2A–2C). Interestingly, even when given 5 hours after MCAO induction, eupatilin (10 mg/kg) significantly reduced tMCAO-induced brain infarction and neurological dysfunction to 26.13% and 16.01% compared with the vehicle-treated group, respectively (Fig 2A–2C; the value of infarcted area and the mNSS in the eupatilin-administered tMCAO group were 23.30% and 11.91 ± 0.54, respectively). These data demonstrate that eupatilin is therapeutically effective in cerebral ischemia. These neuroprotective effects of eupatilin were also confirmed by FJB staining, in which tMCAO challenge caused extensive neural cell death and eupatilin administration reduced it (Fig 2D). The neuroprotective effects of a single administration of eupatilin persisted up to 3 days. Mice were treated with eupatilin (10 mg/kg, *p*.*o*.) once immediately after tMCAO, and brain damages were assessed by FJB staining and the mNSS 3 days after eupatilin administration. Massive cell death was observed 3 days after tMCAO, which was remarkably reduced by eupatilin administration (Fig 3A). Eupatilin also significantly improved the neurological function 3 days after tMCAO (Fig 3B). These data demonstrated that eupatilin administration reduced brain damage after tMCAO in mice.

### Eupatilin reduces microglial activation in tMCAO-challenged brain

Microglial responses have been well characterized in post-ischemic brain, particularly focusing on a time- and region-dependent activation [17,22–24]. Additionally, we here demonstrated *in vitro* anti-inflammatory effects of eupatilin in activated microglia. Therefore, we have assessed whether the observed neuroprotection with eupatilin is associated with the reduction of microglial activation in different regions of ischemic brain by employing immunohistochemical analyses against Iba1, a well-known marker for activated microglia [17,23]. We also assessed the effects of eupatilin on microglial responses at different time points after tMCAO (days 1 and 3 after tMCAO) to verify whether the effects persists to 3 days after tMCAO.

**Fig 4:**
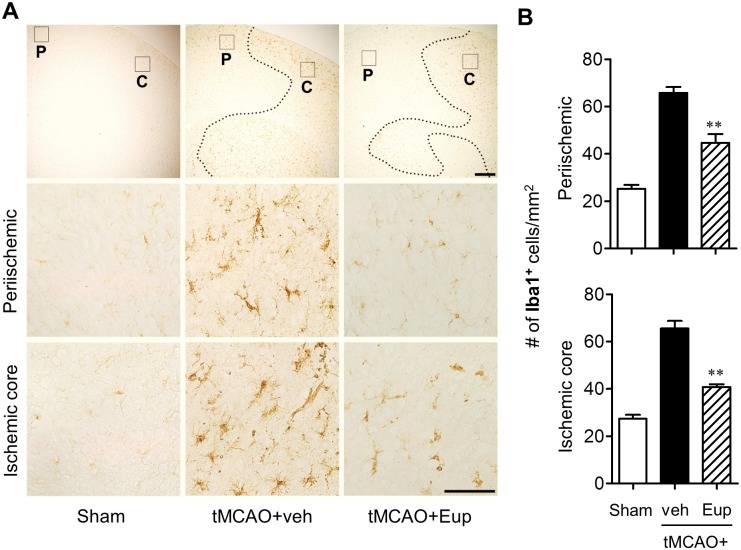
Eupatilin reduces microglial activation in the post-ischemic brain 1 day after tMCAO challenge. Mice were challenged with tMCAO and eupatilin (Eup, 10 mg/kg, *p*.*o*.) was administered immediately after reperfusion. Effects of eupatilin on microglial activation were determined in tMCAO-challenged brain 22 h after reperfusion by immunohistochemistry against Iba1. (**A**) Representative images for Iba1-immunopositive cells in periischemic (‘P’) and ischemic core (‘C’) regions. Diagram boxes in top panels display brain areas where the images in lower panels were acquired. Dashed lines indicate the lesion site. Scale bars, 200 μm (top panels) and 50 μm (middle and bottom panels). (**B**) Quantification of Iba1-immunopositive cells in both regions. n = 4 per group. **P<0.01 versus the vehicle-treated tMCAO group (tMCAO+veh).

**Fig 5:**
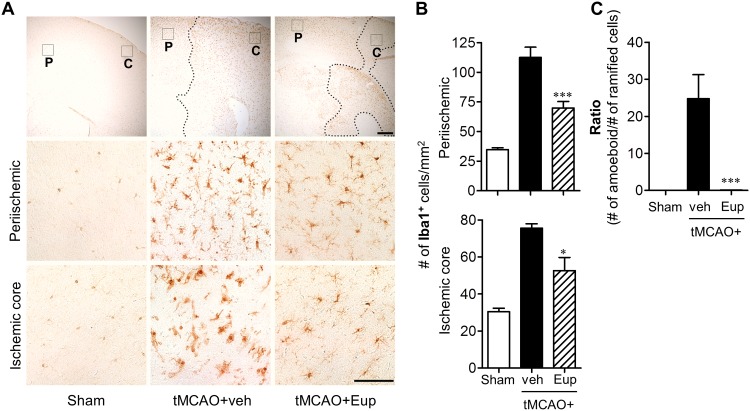
Eupatilin reduces microglial activation in the post-ischemic brain 3 days after tMCAO challenge. Mice were challenged with tMCAO and eupatilin (Eup, 10 mg/kg, *p*.*o*.) was administered immediately after reperfusion. Effects of eupatilin (Eup) on microglial activation and morphological change were assessed in tMCAO-challenged brain 3 days after reperfusion by immunohistochemistry against Iba1. (**A**) Representative images for Iba1-immunopositive cells in periischemic (‘P’) and ischemic core (‘C’) regions. Diagram boxes in top panels display brain areas where the images in lower panels were acquired. Dashed lines indicate the lesion site. Scale bars, 200 μm (top panels) and 50 μm (middle and bottom panels). (**B**) Quantification of Iba1-immunopositive cells in both regions. (**C**) Quantification of morphological changes of Iba1-positive cells in ischemic core region (from ‘ramified’ into ‘amoeboid’ cells) by dividing the number of amoeboid cells with ramified cells. n = 5 per group. *P<0.05 and ***P<0.001 versus vehicle-treated tMCAO (tMCAO+veh).

The number of Iba1-positive cells was significantly increased in both periischemic and ischemic core regions 1 day after tMCAO, which was markedly abolished by eupatilin administration (Fig 4). These effects were more obvious 3 days after tMCAO (Fig 5). Eupatilin reduced the number of Iba1-positive cells in ischemic brain 3 days after tMCAO as well (Fig 5A and 5B). Interestingly, eupatilin induced a dramatic morphological change of activated microglia in ischemic core 3 days after tMCAO induction. In the vehicle-treated group, most of Iba1-positive cells are amoeboid types in the ischemic core region while, in sharp contrast, they were changed into ramified in the eupatilin-administered group (Fig 5A and 5C). These results demonstrated that eupatilin effectively reduced microglial activation in ischemic brain and these effects remained even after 3 days following tMCAO challenge.

**Fig 6:**
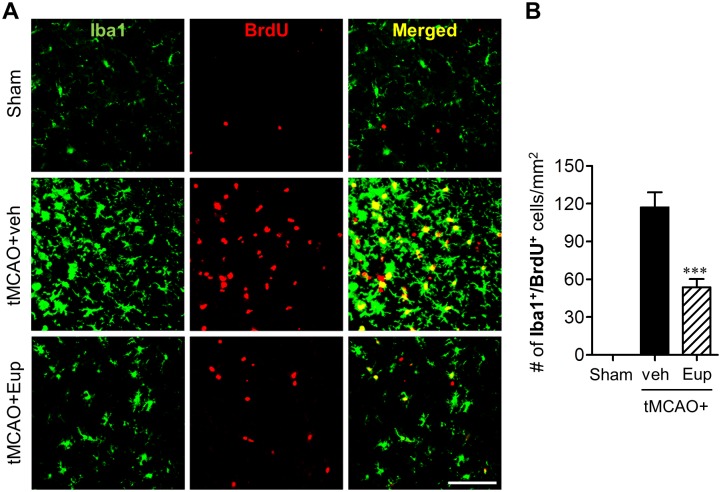
Eupatilin reduces microglial proliferation in the post-ischemic brain 3 days after tMCAO challenge. Mice were challenged with tMCAO and eupatilin (Eup, 10 mg/kg, *p*.*o*.) was administered immediately after reperfusion. Effects of eupatilin (Eup) on microglial proliferation were assessed in tMCAO-challenged brain 3 days after reperfusion by double immunofluorescence against BrdU and Iba1. (**A**) Representative images for BrdU- and Iba1-immunopositive cells in the marginal zone of ischemic brain. Scale bar, 50 μm. (**B**) Quantification of proliferated microglia by counting the number of BrdU/Iba1-double positive cells. n = 5 per group. ***P<0.001 versus vehicle-treated tMCAO (tMCAO+veh).

We further assessed the effects of eupatilin on microglial proliferation in ischemic brain 3 days after tMCAO by double-immunofluorescence against Iba1 and BrdU. The number of Iba1/BrdU double-positive cells was markedly increased in the marginal zone (that is the penumbra) between the periischemic and ischemic core regions (Fig 6), demonstrating microglial proliferation in this area. When mice were treated with eupatilin, the number of double-immunopositive cells was reduced to about 55% (Fig 6). These data demonstrated that eupatilin also effectively suppressed microglial proliferation in ischemic brain.

### Eupatilin reduces lipid peroxidation in tMCAO-challenged brain

**Fig 7:**
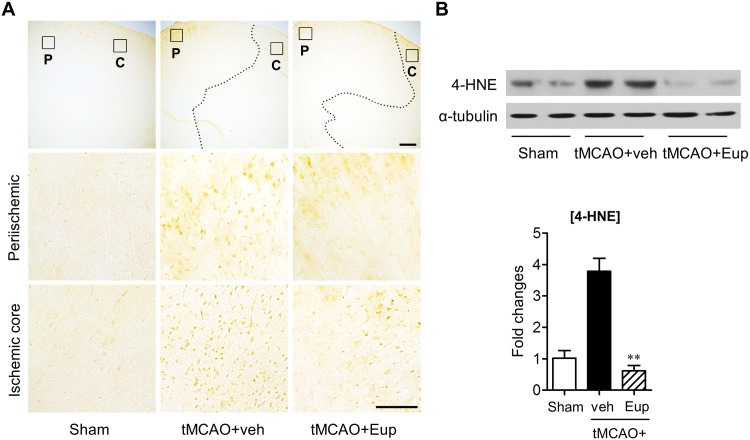
Eupatilin reduces lipid peroxidation in the post-ischemic brain of tMCAO-challenged mice. Mice were challenged with tMCAO and eupatilin (Eup, 10 mg/kg, *p*.*o*.) was administered immediately after reperfusion. (**A**) Effects of eupatilin on lipid peroxidation were determined by immunohistochemistry using an antibody against 4-HNE in tMCAO-challenged brains 22 h after reperfusion. Representative images of 4-HNE-immunopositive cells in periischemic (‘P’) and ischemic core (‘C’) regions. Diagram boxes in top panels display brain areas where the images in lower panels were acquired. Dashed lines indicate the lesion site. Scale bars: 200 μm (top panels), 50 μm (middle and bottom panels). (**B**) Effects of eupatilin (Eup) on 4-HNE production were determined by Western blot in tMCAO-challenged brains 1 day after reperfusion. Changes in 4-HNE and α-tubulin protein levels in the hemisphere with ischemic challenge were shown as representative Western blots and quantification. n = 6 per group. **P<0.01 versus the vehicle-treated tMCAO group (tMCAO+veh).

Oxidative stress is also one of major pathogenetic phenomena during cerebral ischemia, leading to lipid peroxidation [25]. Additionally, eupatilin has been demonstrated *in vitro* as a strong anti-oxidant [7,8], suggesting that the observed neuroprotection is due to, in part, its anti-oxidative property. To determine whether eupatilin reduces lipid peroxidation in tMCAO-challenged brain, the expression level of 4-HNE, a cytotoxic product of lipid peroxidation, was measured using an immunohistochemical analysis. In the vehicle-treated tMCAO group, many cells expressed 4-HNE in both periischemic and ischemic core regions, whereas, as expected, 4-HNE signals in both regions were markedly reduced by eupatilin (Fig 7A). This reduction was confirmed by Western blotting, in which tMCAO challenge caused the upregulation of 4-HNE protein in the ischemic brain and eupatilin significantly attenuated it (Fig 7B). These results demonstrate that eupatilin can alleviate oxidative stress-associated lipid peroxidation in the brain with tMCAO.

### Eupatilin inhibited NF-κB activation in tMCAO-challenged brain

**Fig 8:**
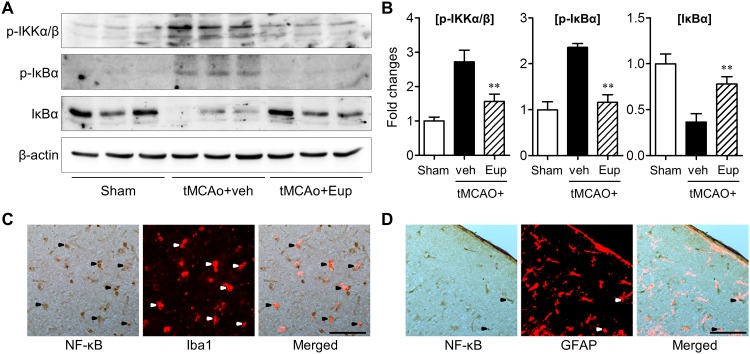
Eupatilin diminishes the activation of NF-κB pathway in the post-ischemic brain of tMCAO-challenged mice. Mice were challenged with tMCAO and eupatilin (Eup, 10 mg/kg, *p*.*o*.) was administered immediately after reperfusion. (**A and B**) Effects of eupatilin (Eup) on NF-κB activation pathway were determined by Western blot in tMCAO-challenged brains 22 h after reperfusion. Changes in p-IKKα/β, p-IκBα, and IκBα protein levels in the hemisphere with ischemic challenge. (**A**) Representative Western blots. (**B**) Quantification. n = 6 per group. **P<0.01 versus vehicle-treated tMCAO (tMCAO+veh). (**C and D**) Localization of NF-kB p65 was determined by double immunolabeling in tMCAO-challenged brains 22 h after reperfusion. (**C**) Representative images of NF-kB p65-immunopositive cells and double-immunopositive cells (NF-kB p65, brown; Iba1, red fluorescence). (**D**) Representative images of NF-kB p65-immunopositive cells and double-immunopositive cells (NF-kB p65, brown; GFAP, red fluorescence). Arrowheads indicate double-immunopositive cells. Scale bar, 50 μm.

NF-κB signaling pathway is well-known to mediate immune responses in ischemic brain [26]. To investigate whether eupatilin inhibits NF-κB pathway, we determined phosphorylation or expression of IKKα/β and IκBα, both of which are regulators of NF-κB signals, in tMCAO-challenged brain. The activation of NF-κB pathway was observed in response to tMCAO, involving phosphorylation of IKKα/β and phosphorylation and degradation of IκBα (Fig 8A and 8B). Eupatilin significantly diminished the phosphorylation of IKKα/β and IκBα and reduced the degradation of IκBα (Fig 8A and 8B). These results demonstrate that eupatilin attenuates NF-κB signaling pathway in ischemic brain. We next determined in which cell types NF-κB activation occurs in the ischemic brain. Glial cells such as microglia and astrocytes are the major immune cells in the CNS [27,28], and they are known to be associated with NF-κB activation [29–31]. Therefore, we determined whether NF-κB p65 subunit is expressed in activated astrocytes and activated microglia 1 day after tMCAO by employing double immunolabeling. NF-κB p65 was abundantly expressed in the ischemic core region, and the signals were localized in most of Iba1^+^ cells, demonstrating that activation of NF-κB pathway in the ischemic brain occurs in activated microglia (Fig 8C). In addition, NF-κB p65 was co-localized to a lesser extent with GFAP, a marker for activated astrocytes, further demonstrating that activation of NF-κB pathway in the ischemic brain also occurs in reactive astrocytes (Fig 8D). These results suggest that microglia inactivation by eupatilin may result in suppression of NF-κB pathway in the ischemic brain.

## Discussion

This study has demonstrated a medically relevant efficacy of eupatilin, an active compound of *A*. Nakai, on transient focal cerebral ischemia, particularly involving its neuroprotective properties on microglial activation. Eupatilin was found *in vitro* to markedly reduce microglial activation, assessed by reduced levels of well-known proinflammatory mediators in activated microglia, such as NO, TNF-α, IL-6, and PGE_2_. In particular, eupatilin was found *in vivo* to attenuate brain damage after transient focal cerebral ischemia, along with reduced microglial activation and proliferation and diminished lipid peroxidation, supporting its neuroprotective potential in cerebral ischemia. It is also of note that eupatilin was neuroprotective against cerebral ischemia upon the administration even after reperfusion (i.e., eupatilin administration immediately after tMCAO or 5 hours after MCAO induction), indicating its therapeutic potential. In addition, activation of NF-κB signaling was associated with the neuroprotective effect of eupatilin in cerebral ischemia.

Eupatilin exerts anti-inflammatory and anti-oxidative properties in diverse *in vitro* and *in vivo* experimental systems [7–10]. Despite these pharmacological properties have been considered to control disease symptoms in various central nervous system (CNS) disorders, little is known on pharmacological effects of eupatilin in the CNS. Previously, we evaluated the neuroprotective effects of eupatilin, in which eupatilin reduced neuronal cell death in the hippocampal CA1 region by enhancing Akt activity in transient global ischemia-challenged brain [14]. This study has demonstrated the therapeutic potential of eupatilin against transient focal cerebral ischemia, modeled with tMCAO. Eupatilin has shown strong neuroprotection, which is closely associated with its beneficial properties on inflammation by regulating activated microglia. Results from these two independent studies suggest that eupatilin could be therapeutically utilized for both types of ischemia, transient global and focal cerebral ischemia. Similarly, there are additional two-relevant reports, supporting its possible efficacy in CNS diseases [13,32]. Stillen^™^ that includes mainly eupatilin was reported to reduce depression-associated behaviors and biochemical mediators, along with reduced inflammatory responses in the hippocampus [13]. In addition, jaceosidin (4’,5,7-trihydroxy-3’,6-dimethoxyflavone), a flavone with a quite similar chemical structure to eupatilin (5,7-dihydroxy-3’,4’,6-trimethoxyflavone), was reported to improve clinical symptoms of experimental autoimmune encephalomyelitis mouse, a model for human multiple sclerosis, *via* inhibiting microglial activation [32]. These independent two studies additionally suggest that eupatilin could be applied to other CNS diseases, particularly neuroinflammation-associated diseases.

Neuroinflammation is a key pathogenesis of cerebral ischemia and characterized by immune responses, likely involving microglial activation [16,18]. In the neuroinflammatory circumstances after ischemia, activated microglia drive a robust production of various neurotoxic molecules including NO and proinflammatory cytokines, leading to brain damage [16,18]. Therefore, how to reduce proinflammatory mediators from activated microglia or how to inactivate microglia has become an important therapeutic strategy against cerebral ischemia [17,18]. So far, anti-inflammatory actions of eupatilin have been well studied, but mostly outside of the CNS. In the CNS, Stillen^™^ reduced the production of IL-1β, IL-6, and TNF-α in the hippocampus [13]. Similarly, in activated microglia, *A*. Nakai extract or jaceosidin was reported to reduce the production of NO and proinflammatory cytokines and associated neuronal death [15,32]. In this study, eupatilin itself reduced proinflammatory mediators including NO, PGE_2_, IL-6, and TNF-α in LPS-stimulated microglia. It is also of note that eupatilin administration reduced microglial activation and proliferation after ischemic challenge. In addition, the effect is further supported by findings on morphological changes of microglia, another prominent feature upon their activation. In normal brain, microglia are shaped as ramified with small soma while, in pathological conditions, they are activated and become rounded amoeboid-like cells, which is well characterized in ischemic brain [17,22,23]. In this study, eupatilin reversed morphological changes of activated microglia from amoeboid-like into ramified cells. These *in vivo* results indicate that eupatilin may exert therapeutic potential against transient focal cerebral ischemia *via* diminishing microglial activation fundamentally.

Oxidative stress is another core pathogenesis of cerebral ischemia, leading to macromolecular peroxidation, apoptotic cascade, inflammatory process, and finally brain damage [25]. The pharmacological property of eupatilin as an anti-oxidant has also been well studied. For example, it protects epithelial cells from oxidative damage by downregulating expression of oxidative-responsible genes with a control of associated signaling pathways [7,8]. We demonstrated that eupatilin reduced lipid peroxidation in ischemic brain, allowing the suggestion that the neuroprotective activity of eupatilin in cerebral ischemia may be due to its anti-oxidant capacity.

NF-κB signaling has been reported as a key pathway for anti-inflammatory efficacy of eupatilin in several immune cells [9,11,33,34]. Eupatilin reduced expression levels of proinflammatory cytokines by blocking NF-κB pathway in LPS-stimulated macrophages [9]. By inhibiting Iκ-Bα degradation and NF-κB promoter activity, eupatilin also exhibited immunosuppressive function in activated T cells [11]. In activated eosinophils and monocytes that contribute to bronchial inflammation, eupatilin reduced immune-associated responses by diminishing NF-κB DNA binding activity, along with reduced phosphorylation of IKKα/β and IκBα [33,34]. It is well-known that the activated NF-κB pathway is a pathogenetic event in response to ischemic challenge [35–37] despite no reported link between eupatilin and this pathway in ischemic brain. Activation of IKK was demonstrated as a key event for neuronal death in ischemic brain, employing conditional IKK nulls in neurons [38] and IκB degradation was diminished with neuroprotective agents in ischemic brain [39]. In this study, eupatilin suppressed IKKα/β phosphorylation, IκBα phosphorylation, and IκBα degradation in ischemic brain, suggesting that its neuroprotective effect may be mediated by inhibiting NF-κB pathway. This eupatilin-driven suppression of NF-κB pathway may come as a result of microglial inactivation by eupatilin because activated microglia are one of main cell types to express NF-κB p65 subunit in the ischemic brain.

Through many independent reports, eupatilin has been identified to reduce inflammatory responses and oxidative stress in non-neural cells. This study revealed that these well-known properties of eupatilin can be applied to transient focal cerebral ischemia, a CNS disorder featured pathologically by neuroinflammation, *via* inhibition of microglial activation. This study also raises the possibility that Stillen^™^, a drug used for treatment of gastric ulcers, is applicable for focal cerebral ischemia, because eupatilin is a main component of this drug although the actual efficacy in cerebral ischemia awaits future evaluations. In addition, the current findings on the neuroprotective effects of eupatilin and relevant mechanisms in cerebral ischemia further indicates the possible therapeutic potential of eupatilin for other types of CNS disorder where microglia-induced neuroinflammation is the major pathogenesis.
